# Heterogeneity of Sensory-Induced Astrocytic Ca^2+^ Dynamics During Functional Hyperemia

**DOI:** 10.3389/fphys.2020.611884

**Published:** 2020-12-10

**Authors:** Kushal Sharma, Grant R. J. Gordon, Cam Ha T. Tran

**Affiliations:** ^1^Department of Physiology and Cell Biology, Center for Molecular and Cellular Signaling in the Cardiovascular System, University of Nevada, Reno School of Medicine, Reno, NV, United States; ^2^Department of Physiology and Pharmacology, School of Medicine, Hotchkiss Brain Institute, University of Calgary, Calgary, AB, Canada

**Keywords:** two-photon imaging, cerebral blood flow, calcium, awake *in vivo*, functional hyperemia, astrocyte

## Abstract

Astrocytic Ca^2+^ fluctuations associated with functional hyperemia have typically been measured from large cellular compartments such as the soma, the whole arbor and the endfoot. The most prominent Ca^2+^ event is a large magnitude, delayed signal that follows vasodilation. However, previous work has provided little information about the spatio-temporal properties of such Ca^2+^ transients or their heterogeneity. Here, using an awake, *in vivo* two-photon fluorescence-imaging model, we performed detailed profiling of delayed astrocytic Ca^2+^ signals across astrocytes or within individual astrocyte compartments using small regions of interest next to penetrating arterioles and capillaries along with vasomotor responses to vibrissae stimulation. We demonstrated that while a 5-s air puff that stimulates all whiskers predominantly generated reproducible functional hyperemia in the presence or absence of astrocytic Ca^2+^ changes, whisker stimulation inconsistently produced astrocytic Ca^2+^ responses. More importantly, these Ca^2+^ responses were heterogeneous among subcellular structures of the astrocyte and across different astrocytes that resided within the same field of view. Furthermore, we found that whisker stimulation induced discrete Ca^2+^ “hot spots” that spread regionally within the endfoot. These data reveal that astrocytic Ca^2+^ dynamics associated with the microvasculature are more complex than previously thought, and highlight the importance of considering the heterogeneity of astrocytic Ca^2+^ activity to fully understanding neurovascular coupling.

## Introduction

Functional hyperemia is a fundamental control mechanism that provides a rapid local increase in blood flow in response to increased neuronal activity. It is well established that neurotransmission can directly affect the vasculature through innervation (Hamel, 2006; Schummers et al., 2008; Nimmerjahn et al., 2009) or through neuromodulators (Bekar et al., 2008; Takata et al., 2011; Ding et al., 2013; Paukert et al., 2014). These processes are critical in cerebral blood flow (CBF) regulation and serve to ensure that the blood supply matches temporally and spatially changing metabolic demands of neurons. It has been proposed that astrocytes are mediators that relay neuronal information to the vasculature – perhaps on slower timescales – helping to control vessel diameter in addition to neurons and, in turn, regulate blood flow (Zonta et al., 2003; Mulligan and MacVicar, 2004; Straub and Nelson, 2007; Gordon et al., 2008). Our previous study has reported that the delayed astrocytic endfoot Ca^2+^ signal is mediated by both neurons and vasculature, suggesting a complex interplay between multiple mechanisms that must temporally and spatially coincide to cause a large activation of endfeet. This intriguing finding necessitates further detailed analysis of endfoot Ca^2+^ dynamics to gain insights into their contributions to functional hyperemia (Tran et al., 2018).

The work performed on *ex vivo* brain slice preparations has shown that increases in cytosolic Ca^2+^ concentration [(Ca^2+^)], produced by uncaging caged Ca^2+^ compounds (Mulligan and MacVicar, 2004; Straub et al., 2006) or through neuronal stimulation (Simard et al., 2003; Zonta et al., 2003; Gordon et al., 2008), are critical mediators of functional hyperemia, suggesting that activity-dependent vascular changes are facilitated by an astrocyte-mediated Ca^2+^-dependent process. Some *in vivo* two-photon imaging studies in anesthetized animals have provided support for this notion, demonstrating rapid astrocytic Ca^2+^ transients followed by vasodilation (Winship et al., 2007; Lind et al., 2013, 2018) or an increase in red blood cell (RBC) velocity (Otsu et al., 2015) in various regions of the cerebral cortex in response to sensory stimuli. However, other *in vivo* studies in anesthetized or slightly sedated animals have provided evidence that functional hyperemia can be achieved in the absence of astrocytic Ca^2+^ increases (Schulz et al., 2012; Takata et al., 2013; Bonder and McCarthy, 2014), or precedes the occasional astrocyte Ca^2+^ transients (Nizar et al., 2013). In awake, resting animals using astrocyte AAV lck-GCaMP6f tools, whisker stimulation triggered both a fast and slow Ca^2+^ signals during functional hyperemia (Stobart et al., 2018a). Our own work in awake and active animals using astrocyte Rhod-2 AM, GCaMP3, or GCaMP6s, revealed that whisker stimulation elicited large astrocytic Ca^2+^ signals that followed rather than preceded vasodilation (Tran et al., 2018). Remarkably, these astrocytic endfoot Ca^2+^ events were mediated by both glutamatergic transmission and vascular-derived nitric oxide. These data signify that astrocytic Ca^2+^ dynamics and its contributions to functional hyperemia maybe more complex than previously thought. It has been shown that astrocytic Ca^2+^ activity is dynamic and heterogeneous (Bindocci et al., 2017; Stobart et al., 2018b). Characterizations of Ca^2+^ activity in astrocytes have commonly focused on the soma, processes, microdomains or macrodomains (Shigetomi et al., 2013; Srinivasan et al., 2015), but rarely on the astrocytic endfoot. This critical subcellular structure of the neurovascular unit has functional relevance to the astro-vascular relationship. Here, we characterized the cortical astrocyte Ca^2+^ dynamics, in particular astrocytic endfoot Ca^2+^, and examined its relationship with functional hyperemia in completely awake mice *in vivo* using two-photon imaging.

## Materials and Methods

### Animals

The Animal Care and Use Committee of the University of Calgary approved all the animal procedures. All studies were either performed on male GLAST-Cre ERTx LSL-GCaMP3 mice (Jax#014538) between postnatal day 30 (P30) and P60. Animals were injected on three consecutive days with tamoxifen (100 mg/kg, Sigma), prepared as a 10 mg/ml stock in corn oil. Injections started between P19 and P35. Animals were kept on a normal 12-h light/12-h dark cycle and had *ad libitum* access to food and water.

### Awake *in vivo* Preparation

All surgical procedures and isoflurane anesthesia were performed as previously described (Tran and Gordon, 2015). Briefly, 1 week before the imaging session, a head bar was surgically installed on the animal, after which the animal was returned to its home cage to recover. Mice were initially trained on a passive air-supported Styrofoam ball treadmill under head restraint for 30 min and habituated to whisker stimulation with an air puff on contralateral vibrissae once every minute for 5 s using a Picospritzer III (General Valve Corp.) for 2 consecutive days. After training, the animal was returned to its home cage. On imaging day, bone and dura over the primary somatosensory cortex were removed and a ∼3 × 3mm cover glass (thickness #0) was installed over the cranial window.

### Vessel Indicators

Rhodamine B isothiocyanate (RhodB)-dextran (MW 70,000; Sigma) was injected *via* the tail vein (100–200 μl of a 2.3% (w/v) solution in saline) to visualize the blood plasma. The animal was allowed to recover on the treadmill, with its head immobilized, for 30 min prior to imaging.

### Two-Photon Fluorescence Imaging and Whisker Stimulations

Fluorescence images were obtained using a custom-built *in vivo* two-photon microscope (Rosenegger et al., 2014) illuminated with a tunable Ti:sapphire laser (Coherent Chameleon, Ultra II), equipped with GaAsP PMTs (Hamamatsu) and controlled by an open-source ScanImage software. A Nikon 16X objective lens (0.8NA, 3 mm WD) or a Zeiss 40X objective lens (1.0NA, 2.5 mm WD) was used. GCaMP3 and Rhodamine B dextran were excited at 920 nm. Green fluorescence signals were obtained using a 525/50 nm band-pass filter, and an orange/red light was obtained using a 605/70 nm band-pass filter (Chroma Technology). Bidirectional xy raster scanning was used at a frame rate of 3.91 Hz. Animal behaviors were captured using a near-infrared LED (780 nm) and a camera at 14 Hz. A 5-s air puff that deflected all whiskers on the contralateral side without impacting the face was applied using a Picospritzer while vasodilation and astrocytic Ca^2+^ responses were monitored in the barrel cortex (layers 1–3).

### Data Analysis and Statistics

All data were processed using ImageJ. Movement artifacts in the xy plane were corrected for using the align_slices plugin. ROIs corresponding to astrocyte endfeet, soma, and arbor were analyzed separately. Small ROIs (2.5×2.5 μm) placed next to one and another around the endfoot was analyzed to obtain temporal sequence of Ca^2+^ signals around the endfoot. Ca^2+^ responses were calculated as ΔF/F = (F_t_−F_rest_)/F_rest_, where F_t_ is the measured fluorescence at any given time and F_rest_ is the average fluorescence obtained over 2 s prior to whisker stimulation. Ca^2+^ signals with an intensity that crossed a 3-standard deviation (SD) threshold (average 3SD: ΔF/F_ef_ = 5.9; ΔF/F_soma_ = 3.8; ΔF/F_arbor_ = 2.6 and the associated coefficient of variation: 56.5, 65.6, and 63.5% respectively) relative to signal fluctuations during a 2-s prestimulus baseline and remained above the threshold for at least 0.5 s were detected as astrocyte Ca^2+^ increases. Penetrating arteriole cross-sectional area was analyzed by using the threshold feature in imageJ, after which particle analysis was used to measure the area of the lumen filled with RhodB-dextran. Cross-sectional area changes were calculated as Δd/d = (d_t_−d_rest_)/d_rest_ where d_t_ is the area obtained at any given time and d_rest_ is the average baseline area obtained over 2 s prior to whisker stimulation. Area change with an intensity that crossed a 3-SD threshold (average SD for Δd/d = 2.8; coefficient of variation: 50.8%); relative to signal fluctuations during a 2-s prestimulus baseline, and remained above the threshold for at least 0.5 s were detected as vasodilation. Onset corresponds to the first time point at which the signal reached the threshold and remained over it for at least 0.5 s. Duration was calculated as the difference between response onset and response offset. Statistical analyses used a paired or unpaired *t*-test or one-way analysis of variance (ANOVA) followed by Tukey’s multiple comparisons test as appropriate. Statistical “n” constituted a single experimental trial or an experimental animal, as indicated. Data are expressed as means ± SEM. values of *p* <0.05 were considered statistically significant. All statistical analyses were done using GraphPad. A 95% confidence interval (CI) was calculated using modified Wald method.

## Results

### Contralateral Whisker Stimulation Predominantly Elicits Reliable Arteriolar Dilation, but Generates Heterogeneous Astrocytic Ca^2+^ Responses

**Figure 1:**
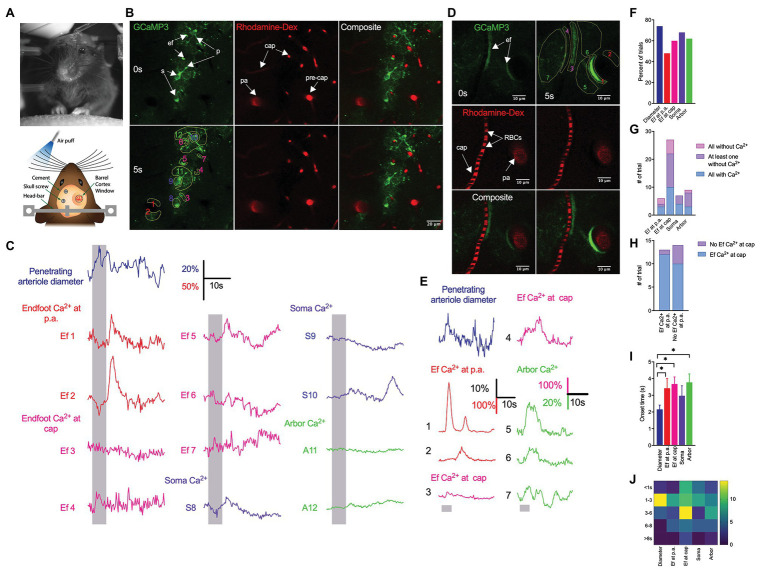
Contralateral whisker stimulation predominantly elicits reliable arteriole dilation but generates heterogeneous astrocytic Ca^2+^ responses. **(A)** Image of an awake mouse and schematics showing the *in vivo* experimental setup. **(B)** Representative images from a cranial window over the barrel cortex of a GLAST-Cre ERT x GCaMP3 mouse showing Ca^2+^ fluctuations (green) from endfoot (ef), soma (s), process (p) and vessel response in penetrating arterioles (pa), pre-capillaries (pre-cap) and capillaries (cap) [labeled with Rhod-B dextran (red)] before (top images) and during (bottom images) a 5 s whisker stimulation. Regions of interest (ROIs) (bottom left) correspond to representative traces in **(C)**. **(C)** Representative traces from a GLAST-Cre ERT x GCaMP3 mouse (from ROIs as indicated in **B**) showing the percent change in peak diameter of a penetrating arteriole (navy) and Ca^2+^ activity from an endfoot at the penetrating arteriole (red), endfoot at the capillaries (magenta), soma (purple), and arbor (green) in response to a 5-s whisker stimulation. Vertical shaded areas indicate periods of whisker stimulation. **(D)** Higher-magnification images of a penetrating arteriole and nearby capillary enveloped by an astrocytic endfoot from a different mouse showing responses immediately prior to (0 s) and during (5 s) whisker stimulation. ROIs (top right) correspond to representative traces in **(E)**. **(E)** Representative traces showing changes in penetrating arteriole diameter change and astrocytic Ca^2+^ fluctuations (from ROIs as indicated in D (top right)) in different subcellular compartments in response to whisker stimulation. **(F)** Plot showing the percentage of trials generating arteriole dilation and Ca^2+^ increases in response to a 5-s whisker stimulation. **(G)** Summary showing the number of trials that displayed Ca^2+^ changes in all, all but one (at least one) or none from a given subcellular structure among several astrocytes observed within a single field of view. **(H)** Summary showing heterogeneity of Ca^2+^ increases observed at endfeet wrapping around the penetrating arterioles and those enwrapping capillaries when they were imaged simultaneously. **(I)** Summary showing arteriole dilation onset time and that of Ca^2+^ changes in all subcellular compartments of the astrocyte in response to a 5 s whisker stimulation. **(J)** Heat map showing the distribution of onset time of arteriole dilation and Ca^2+^ changes in all subcellular compartments of the astrocyte.

We previously showed that sensory stimulation induces rapid functional hyperemic response that is followed by delayed astrocytic Ca^2+^ (Tran et al., 2018). In this previous study, we primarily focused on a single penetrating arteriole enwrapped by an endfoot and associated soma and arbor. Since other astrocytes, and in particular endfeet, that enwrapped nearby capillaries within the same cortical layer as that of penetrating arterioles were not examined, it remained unclear whether sensory stimulation induced a global effect that elicited homogeneous Ca^2+^ changes in all astrocytes. In the present study, we extended these analyses, examining Ca^2+^ dynamics in as many astrocytes within the field of view as possible and monitoring endfoot Ca^2+^ changes associated with penetrating arterioles and capillaries in the same cortical layer and focal plane. We used a genetically engineered Ca^2+^ indicator, a cytosolic form of GCaMP3, driven by the tamoxifen-inducible astrocyte-specific promoter, *Slc1a3*-Cre/ERT (GLAST-ERT), to assess local intracellular astrocyte Ca^2+^ dynamics, with concurrent tail vein injection of RhodB-dextran to visualize the vasculature and monitor vascular responses (Figures 1A,B,D). A 5-s whisker stimulation of contralateral side vibrissae predominantly induced a rapid functional hyperemic response that was followed by a rise in astrocytic Ca^2+^ (Figures 1C,E,G, *n* = 9 mice; number of trials: vessels, 25; endfoot at penetrating arteriole, 33; endfoot at capillaries, 68; soma, 28; arbor, 37). Even though stimulation of vibrissae did not elicit arteriole dilation in all cases, it induced more vasodilatory responses (74%, CI: 0.55–0.87) than Ca^2+^ rises (48%, CI: 0.33–0.65) in endfeet, enveloping the penetrating arterioles (Figure 1F). Sensory-induced increases in Ca^2+^ rises were observed in some, but not all, soma (68%, CI: 0.49–0.82), endfeet (at cap: 60%, CI: 0.48–0.71) and arbors (62%, CI: 0.46–0.76; Figure 1F). Interestingly, there were more cases where sensory-associated Ca^2+^ increases were observed at endfeet wrapping around the capillaries than at those enwrapping the penetrating arterioles (Figure 1F). More importantly, the spatial and temporal profiles of Ca^2+^ signals in a given subcellular structure were not always similar from one astrocyte to another when they were imaged simultaneously within the same field of view (Figures 1G–K). While whisker stimulation elicited a rise in astrocytic Ca^2+^ in all regions of interest (ROIs) from the subcellular compartments in some trials (endfoot at penetrating arterioles: three trials, CI: 0.18–0.81; endfoot at capillaries: 10 trials, CI: 0.23–0.59; soma: four trials, CI: 0.24–0.84; arbor: three trials, CI: 0.12–0.65), it did not trigger Ca^2+^ changes in any ROIs in other trials. There were also trials in which at least one ROI did not exhibit a rise in Ca^2+^ (Figure 1G). An increase in endfoot Ca^2+^ associated with penetrating arterioles was not always accompanied by an increase in endfoot Ca^2+^ at the capillaries and similarly, a lack of endfoot Ca^2+^ increases at the penetrating arterioles did not always correspond to a lack in endfoot Ca^2+^ changes at the capillaries when they were imaged simultaneously within the same field of view (an increase in endfoot Ca^2+^ at both penetrating arterioles and capillaries: 12 trials, CI: 0.65– >0.99; no increase in endfoot Ca^2+^ at penetrating arterioles with an increase in endfoot Ca^2+^ at capillaries: 10 trials, CI: 0.45–0.89; Figure 1H). Functional hyperemic responses were not only more frequently observed than Ca^2+^ rises, but were also initiated before endfoot Ca^2+^ increased (onset: 2.2 ± 1.1 s and 3.4 ± 2.3 s for diameter and endfoot Ca^2+^ response respectively; *p* = 0.02; Figure 1I), a finding that is consistent with our previous observation (Tran et al., 2018). Although the onset of functional hyperemia was faster than that of endfoot, soma or arbor Ca^2+^ increases, the durations and time to peak of all responses were comparable (Data not shown). The distribution of onset time for sensory-induced astrocytic Ca^2+^ changes was more widespread than that of functional hyperemic responses (Figure 1J). While the majority of trials displayed an onset time between 1 and 3 s for functional hyperemia, the onset time of astrocytic Ca^2+^ rise could be varied from less than 1 s to more than 8 s (Figure 1J).

### Whisker Stimulation-Induced Arteriole Dilation Occurs in the Absence of Astrocytic Endfoot Ca^2+^

**Figure 2:**
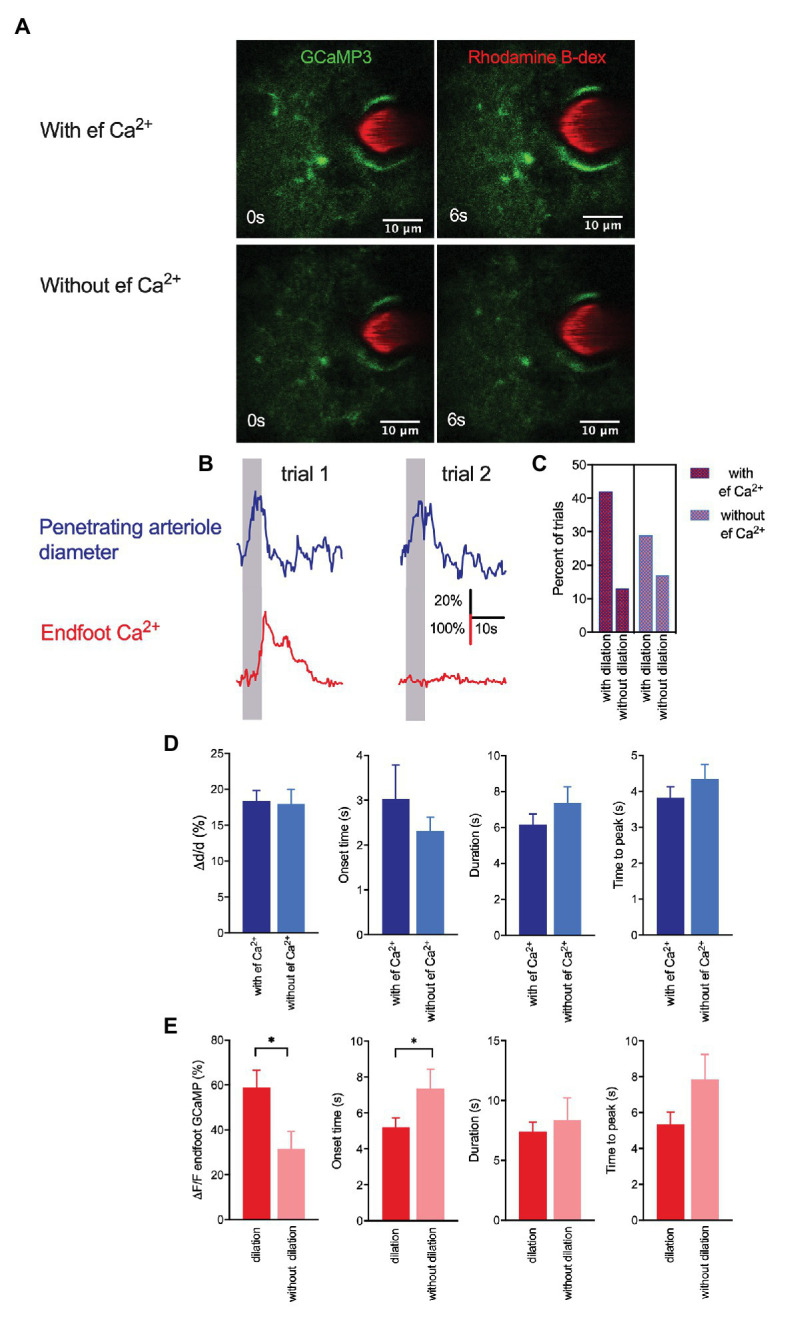
Whisker stimulation-induced arteriole dilation occurs in the absence of astrocytic endfoot Ca^2+^ but vasodilation augmented endfoot Ca^2+^. **(A)** Representative images from two different trials showing a penetrating arteriole enwrapped by an endfoot of a GLAST-Cre ERT x GCaMP3 mouse immediately prior to (0 s) and in response to (6 s) whisker stimulation. **(B)** Representative traces showing penetrating arteriole diameter and changes in endfoot Ca^2+^ (from **A**) from the same mouse but different trials in response to a 5-s whisker stimulation. **(C)** Percentage of trials with and without dilation in the presence and absence of endfoot Ca^2+^. **(D)** Summary of percent peak change (i), onset time (ii), duration (iii), and time to peak (iv) of penetrating arteriole diameter in response to whisker stimulation in the presence or absence of endfoot Ca^2+^. **(E)** Summary of percent peak change (i), onset time (ii), duration (iii) and time to peak (iv) of endfoot Ca^2+^ in response to whisker stimulation in the presence or absence of vasodilation.

Although some *in vivo* studies have reported that arteriole dilation is associated with a rapid rise in endfoot Ca^2+^ (Lind et al., 2013, 2018; Stobart et al., 2018a), others have shown that a majority of vasodilatory responses to sensory stimulation lack an associated astrocytic Ca^2+^ response (Nizar et al., 2013; Bonder and McCarthy, 2014). In the present work, vasodilation in response to a 5-s whisker stimulation was observed in both the presence and absence of a rise in endfoot Ca^2+^ (Figures 2A,B). Of the total 175 trials in 22 mice, 95 exhibited an endfoot Ca^2+^ increase and 80 lacked it. Trials with a rise in endfoot Ca^2+^ were more frequently associated with vasodilation (42%) than those without an endfoot Ca^+^ increase (29%; Figure 2C), but we found no significant differences in peak percentage increase in arteriole cross-sectional area, onset time or duration of dilation, or time to peak dilation between these two trial groups (Figure 2D). These data suggest that changes in local endfoot Ca^2+^ are not directly linked to arteriole dilation during experimentally evoked sensory stimulation in completely awake behaving mice. On the other hand, arteriole dilation significantly enhanced endfoot Ca^2+^ (ΔF/F = 58.9 ± 7.8% with vasodilation vs. 31.6 ± 7.7% without vasodilation; *p* = 0.02, *n* = 22 mice, 175 trials; Figure 2E). Furthermore, the onset of endfoot Ca^2+^ elevations was significantly faster in cases when there was an associated vasodilation (5.2 ± 0.5 s vs. 7.4 ± 1.1 s, *p* = 0.04, *n* = 22 mice, 175 trials). These observations further support our previous findings that changes in arteriole diameter evoked endfoot Ca^2+^ transients (Tran et al., 2018).

### Whisker Stimulation Elicits Discrete Endfoot Ca^2+^ Signals That Subsequently Spread

**Figure 3:**
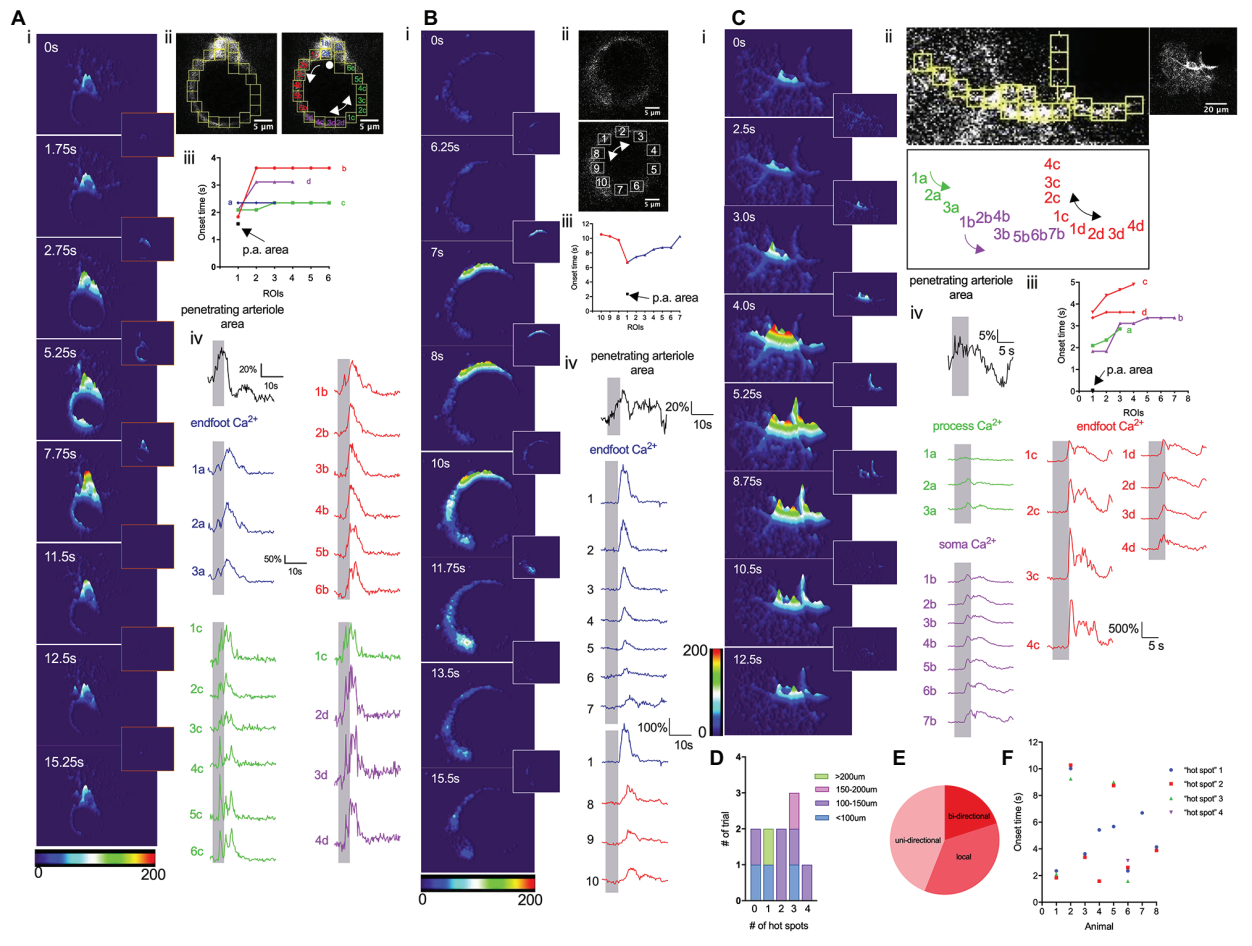
Whisker stimulation elicits discrete endfoot Ca^2+^ signals that subsequently spread. **(A)** Detailed analysis of Ca^2+^ dynamics in response to a 5 s whisker stimulation. (i). 3-D surface plot of astrocyte Ca^2+^ fluctuations in an astrocyte at different time points immediately prior to (0 s), during (1.75 s, 2.75 s), or after (5.25 s, 7.75 s, 11.5 s, 12.5 s, and 15.25 s) a 5-s whisker stimulation. Insets showed the differential images between the two time points. (ii) Higher magnification image of an endfoot and consecutive ROIs from which Ca^2+^ measurements were obtained. Double-headed arrow indicates the bidirectional spread of the Ca^2+^ “hot spot” spread. Single-headed arrow indicates the uni-directional spread of the Ca^2+^ “hot spot”. A dot indicates Ca^2+^ signals remain localized. (iii) Onset times from different ROIs showing regional Ca^2+^ activity and vasodilation. (iv) Penetrating arteriole cross-sectional area trace (black) and Ca^2+^ traces (colors) from different ROIs. Vertical shaded areas indicate periods of whisker stimulation. **(B)** Detailed analysis similar to that in A, but from a different animal displaying only the endfoot enwrapping a penetrating arteriole. (i) 3-D surface plot of astrocyte Ca^2+^ fluctuations at different time points immediately prior to (0 s) and after (6.25 s, 7 s, 8 s, 10s, 11.75 s, 13.5 s, and 15.5 s) a 5 s whisker stimulation. Insets showed the differential images between the two time points. (ii) Higher-magnification image of the endfoot and consecutive ROIs (right) from which Ca^2+^ measurements were obtained. Double-headed arrow indicates bidirectional spread of the Ca^2+^ “hot spot”. (iii) Onset times from different ROIs showing endfoot Ca^2+^ activity and vasodilation. (iv) Penetrating arteriole cross-sectional area trace (black) and Ca^2+^ traces (colors) from different ROIs. Vertical shaded areas indicate periods of 5 s whisker stimulation periods. **(C)** Detailed analysis similar to that in **(A)** and **(B)**, but from a different animal showing all subcellular compartments of an astrocyte. (i) 3-D surface plot showing astrocytic Ca^2+^ fluctuations at different time points immediately prior to (0 s), during (2.5 s, 3 s, and 4 s) and after (5.25 s, 8.75 s, 10.5 s, and 12.5 s) a 5 s whisker stimulation. Insets showed the differential images between the two time points. (ii) Higher-magnification image of an endfoot and consecutive ROIs (top left) from which Ca^2+^ measurements were obtained. Labels of all the ROIs (bottom) indicated previously. Double-headed arrow indicates bidirectional spread of the Ca^2+^ “hot spot”. Lower magnification image of the entire astrocyte (top right). (iii) Onset times from different ROIs showing regional Ca^2+^ activity and vasodilation. (iv) Penetrating arteriole cross-sectional area trace (black) and Ca^2+^ traces (colors) from different subcellular compartments. Vertical shaded areas indicate periods of whisker stimulation. **(D)** Summary showing the number of “hot spots” detected associated with the area of the endfoot. **(E)** Pie chart showing the percentage of how those “hot spots” spread. **(F)** Scatter plot of onset time for each “hot spot” from individual mouse.

We next performed a detailed analysis of endfoot Ca^2+^ dynamics in response to a 5-s whisker stimulation. Previous studies have shown that astrocytic Ca^2+^ activity is heterogeneous across different subcellular structures within an astrocyte (Bindocci et al., 2017). Under basal conditions, gliapil, the peripheral region of an astrocyte composed of fine processes, exhibit the highest activity, whereas, soma exhibit the lowest activity. Given their close proximity to the vessel wall, endfeet could conceivably directly modulate, or be modulated by, the vasculature. A detailed analysis of Ca^2+^ activity within a cross-section of an endfoot enwrapping a penetrating arteriole that dilated in response to whisker stimulation revealed heterogeneous Ca^2+^ signals (Figure 3). The discrete nature of these Ca^2+^ signals are revealed in 3-D surface plots that also display, once initiated, how these Ca^2+^ signals spread (Figures 3Ai,Bi,Ci). Subsequently, we analyzed Ca^2+^ responses in small regions of interest (ROIs) positioned next to one and another around the endfoot (Figures 3A,B,Cii-iv). Interestingly, sensory stimulation did not initiate a global rise in endfoot Ca^2+^; instead, it triggered Ca^2+^ “hot spots” at various discrete regions of the endfoot, from which Ca^2+^ signals then spread either bidirectionally or unidirectionally (Figure 3E). While some endfeet had several Ca^2+^ “hot spots” with different onsets (Figures 3A,F), others had only a single hot spot (Figures 3B,F). Occasionally, endfoot Ca^2+^ increases were observed without any discernable “hot spots” (Figure 3D; *n* = 10 animals). In some instances, a soma appeared to be physically part of the endfoot; in this particular scenario, Ca^2+^ signals increased within the soma region either remained localized or did not spread far (Figure 3Ai). This behavior is similar to that observed by Bindocci and colleagues under basal conditions where Ca^2+^ signals were relatively confined to the boundary of the soma (Bindocci et al., 2017). In another instance in which we could clearly see the endfoot, its associated processes and a soma, 3-D surface plots revealed the spread of Ca^2+^ signals from different hot spots within each subcellular structure of the astrocyte. However, signals from these astrocytic compartments appeared to be independent of each other (Figure 3C).

## Discussion

In this study, we revealed that (1) sensory stimulation did not generate a global effect that elicited homogeneous Ca^2+^ changes in all astrocytes or subcellular compartments of an astrocyte; (2) although the absence of endfoot Ca^2+^ around the penetrating arteriole did not preclude arteriole dilation, stronger endfoot Ca^2+^ rises were observed in the presence of vasodilation; (3) sensory stimulation did not elicit a global rise in endfoot Ca^2+^, but instead triggered discrete Ca^2+^ “hot spots” that typically spread around the endfoot and occasionally remained localized. These findings indicate that astrocytic Ca^2+^ dynamics are heterogeneous across different astrocytes as well as between astrocytic subcellular compartments, and suggest that these Ca^2+^ signals may be compartmentalized during sensory-induced functional hyperemia. They further suggest that the close proximity between the endfoot and the vessel wall of a penetrating arteriole does not necessarily translate to direct effects of local endfoot Ca^2+^ on arteriole dilation.

In the past decade, studies performed *in vivo* have presented polarized views on the involvement of astrocytic Ca^2+^ in functional hyperemia. Some of these studies in anesthetized or slightly sedated animals have shown that fast astrocytic Ca^2+^ transients precede functional hyperemic responses to sensory stimulation (Takano et al., 2006; Winship et al., 2007; Petzold et al., 2008; Lind et al., 2013, 2018; Stobart et al., 2018a), whereas others have shown that such signals are absent (Schummers et al., 2008; Schulz et al., 2012; Nizar et al., 2013; Bonder and McCarthy, 2014). Our earlier *in vivo* studies in awake active mice in which we focused on dynamic interactions between a small region of a single penetrating arteriole and its associated endfoot using cross-section imaging showed that a 5-s whisker stimulation induced a rapid functional hyperemic response followed by a delayed increase in endfoot Ca^2+^ (Tran et al., 2018). The current study in awake animals not only showed that vibrissae stimulation produced fast vasodilation followed by less reliable astrocytic Ca^2+^ increases but it also revealed the heterogeneous characteristic of astrocytic Ca^2+^ signals across different astrocytes within a single field of view (Figure 1). These whisker stimulation-induced Ca^2+^ signals appeared to be asynchronous and regional. Our findings are in agreement with previous studies (Bindocci et al., 2017; Stobart et al., 2018b) that demonstrated some fundamental differences in Ca^2+^ dynamics between individual structures of an astrocyte. These observations implicate a compartmentalizing effect in astrocyte Ca^2+^ dynamics. They also call attention to the fact that the majority of prior *in vivo* studies monitored Ca^2+^ changes selectively at small regions of astrocytic subcellular structures or assessed bulk changes in Ca^2+^ in the entire astrocyte, but nonetheless concluded that these observations were representatives of the whole-cell activity or the activity of the entire network. In the current study, simultaneous measurements of vascular reactivity and astrocytic Ca^2+^ changes revealed that continuous vibrissae stimulation for 5 s predominantly induced arteriole dilation that was accompanied by a rise in endfoot Ca^2+^. However, a significant number of trials showed vasodilation in the absence of endfoot Ca^2+^ change, a finding in agreement with previous studies (Schulz et al., 2012; Nizar et al., 2013; Bonder and McCarthy, 2014). Nevertheless, our data showed the likelihood of observing vasodilation with endfoot Ca^2+^ vs. that without endfoot Ca^2+^ was ~42% vs. 29% instead of 10% vs. 90%, as reported from Nizar and colleagues (Nizar et al., 2013). They are also clearly distinct from a previous report that endfoot Ca^2+^ responses were completely absent in all trials (Bonder and McCarthy, 2014). These discrepancies could be attributable partly to the use of anesthesia (Thrane et al., 2011). These findings do not necessarily refute the role of endfoot Ca^2+^ in regulating functional hyperemia on a whole. In fact, they highlight the heterogeneity of astrocytic Ca^2+^ dynamics and suggest that the close proximity of the endfoot and vessel wall does not universally translate to a direct effect of endfoot Ca^2+^ changes on local vasodilation. The dilation of penetrating arterioles observed in layers I–III of the cortex reported here could be due to the retrograde vascular conduction that was initiated deep in the cortex (Uhlirova et al., 2016; Longden et al., 2017), and the endfoot Ca^2+^ increases subsequently observed in deeper cortical layers could exhibit different dynamics from those observed in the relatively superficial layer of the cortex. Interestingly, we found here that sensory-induced endfoot Ca^2+^ increases were significantly stronger when they were accompanied by vasodilation of the penetrating arterioles (Figure 2), further supporting our previous observations that vasodilation obtained independent of neural activity modulates endfoot Ca^2+^ (Tran et al., 2018). The changes in astrocytic Ca^2+^ in response to changes in vasomotor tone suggest a potential arteriole-to-astrocyte communication as previously suggested by our work and others (Kim et al., 2016; Tran et al., 2018).

Situated as they are between neurons and the vasculature, forming a tripartite architecture, astrocytes are ideally positioned to facilitate neuron-vascular communication and have been increasingly viewed as a hub of integrated activity that modulates neuronal and vascular responses. Activity-dependent astrocytic Ca^2+^ responses are typically reported as global events (Zonta et al., 2003; Lind et al., 2013), in which increased neuronal activity triggers a global rise in Ca^2+^ throughout the whole astrocyte or individual compartment. A recent work described more localized events that spread within the confined boundaries of the subcellular structures (Bindocci et al., 2017). Similar to these latter studies, our work reported here using cross-section imaging explored Ca^2+^ dynamics in some of the endfeet that almost completely ensheathed the vessel (Figure 3). Although an initial analysis appeared to show a global rise in endfoot Ca^2+^ in response to a 5-s whisker stimulation; a detailed analysis of these Ca^2+^ responses revealed discrete Ca^2+^ “hot spots” that typically spread. The majority of these “hot spots” spread uni-directionally, while others spread bidirectionally around the endfoot. Yet, in some instances, the “hot spot” remained as a single discrete Ca^2+^ signal. There were also cases where no discernible “hot spot” was observed. In agreement with a previous work (Bindocci et al., 2017), we found that astrocytic Ca^2+^ signals were compartmentalized. For example, in cases where the soma was a part of an endfoot, the somatic Ca^2+^ signal seemed to remain confined to the region defined as a soma. Similar observations were noted in other soma that were not physically a part of the endfoot. These findings suggest that astrocyte can assemble as multiple local units that function heterogeneously, implying that astrocytes can locally sense features of their surrounding environment, whether it is a nearby neuron or a vessel wall, and respond accordingly. Studies have further shown that resting Ca^2+^ differs in different astrocytic regions (Zheng et al., 2015), suggesting that there might be some regional control. These observations suggest that there are specific regions within the soma, endfoot and process where plasma membrane channels (e.g., transient receptor potential vanilloid 4, TRPV4) and/or intracellular organelles possessing the machinery, such as the mitochondria (Agarwal et al., 2017), to initiate Ca^2+^ changes reside, and that these are responsible for inducing the spread of Ca^2+^ signals, perhaps *via* Ca^2+^-induced Ca^2+^ release. Similarly, these subcellular structures may have organelles, such as endoplasmic reticulum (ER) or mitochondria that buffer Ca^2+^ and prevent the signals from spreading beyond a boundary. Other studies have described global events observed in all subcellular compartments (Ding et al., 2013; Paukert et al., 2014; Srinivasan et al., 2015). However, these events appeared to be associated with specific physiological conditions, in this case, a startle response (Ding et al., 2013; Bonder and McCarthy, 2014; Paukert et al., 2014; Srinivasan et al., 2015). This implies that an astrocyte needs to reach a certain threshold before it can trigger the coordination between different compartments and, potentially, between different astrocytes. In the quiescent state, astrocytes have a high threshold for activation such that other systems, such as noradrenergic (Paukert et al., 2014) and/or cholinergic (Takata et al., 2011) circuits, must be recruited to enhance local signals.

Our work has unveiled the complexity of astrocytic Ca^2+^ dynamics and addressed the relationship between such Ca^2+^ signals and NVC. We do not dispute the role of astrocytic Ca^2+^ in regulating vascular response; however, for several reasons, we recommend caution in universally interpreting NVC based on data from an isolated subpopulation of astrocytes imaged at a certain layer of the cortex. First, since astrocytic Ca^2+^ activity is heterogeneous, it would be inaccurate to take observations from a single compartment or a single astrocyte as being representative of the whole cell or the whole network. Second, somatosensory cortical activation is significantly different between layers of the cortex (Krupa et al., 2004). Finally, different levels of the vascular network are structurally and functionally reflected in differences in architecture (Shih et al., 2015), anastomoses, level of neuronal innervation (Hamel, 2006), and expression of receptors and ion channels (Sercombe et al., 1990). Collectively, this vascular architecture serves to provide a supply of blood to every single neural cell sufficient to match the metabolic needs of the cell. In fact, recent studies have demonstrated temporal differences in sensory-induced vasodilation across different layers of the cortex, with the fastest onset of dilation observed below layer IV (Uhlirova et al., 2016). It has been proposed that upstream penetrating arterioles or pial arterioles dilate in response to signals initiated few 100 μm away achieved *via* a retrograde vascular conduction mechanism (Longden et al., 2017). Nevertheless, this does not imply that conduction is the only process that mediates upstream dilation nor does it rule out the possible involvement of NVC at all cortical depths. There are some important caveats that we need to acknowledge: (1) all two-photon fluorescence imaging were conducted at a frame rate of 3.91 Hz. This might have prevented us from detecting faster discrete Ca^2+^ signals; (2) we acknowledge that the fact that small ROIs used to detect discrete Ca^2+^ were done manually could contribute to the under-detection of the signals. In conclusions, our findings further emphasize that examining NVC in 3-Ds and at different layers of the cortex in awake animals is necessary to obtain accurate assessments of astrocytic Ca^2+^. Furthermore, they highlight the need to have an automation of Ca^2+^ analysis designed to better detect all signals from all subcellular compartments of the astrocyte.

## Data Availability Statement

The original contributions presented in the study are included in the article/supplementary materials, further inquiries can be directed to the corresponding author.

## Ethics Statement

The animal study was reviewed and approved by The Animal Care and Use Committee of the University of Calgary.

## Author Contributions

CT and GG: conceptualization, methodology, writing – review and editing. CT: investigation. CT and KS: formal analysis, writing – original draft. All authors contributed to the article and approved the submitted version.

### Conflict of Interest

The authors declare that the research was conducted in the absence of any commercial or financial relationships that could be construed as a potential conflict of interest.
